# Paleoproteomic identification of the species used in fourteenth century gut-skin garments from the archaeological site of Nuulliit, Greenland

**DOI:** 10.1038/s41598-024-63243-0

**Published:** 2024-06-11

**Authors:** Annamaria Cucina, Anne Lisbeth Schmidt, Fabiana Di Gianvincenzo, Meaghan Mackie, Carla Dove, Aviâja Rosing Jakobsen, Bjarne Grønnow, Martin Appelt, Enrico Cappellini

**Affiliations:** 1 Department of Chemical Sciences, Organic Mass Spectrometry Laboratory, University of Catania, Via S. Sofia 64, 95123 Catania, Italy; 2 CQRC- Ospedali Riuniti Villa Sofia- Cervello, Palermo, Via del Vespro 133, 90127 Palermo, Italy; 3 The National Museum of Denmark, Research, Collections and Conservation, I.C. Modewegsvej, 2800 Kgs Lyngby, Denmark; 4 Globe Institute, University of Copenhagen, Øster Farimagsgade 5, 1353 Copenhagen K, Denmark; 5 Faculty of Chemistry and Chemical Technology, University of Ljubljana, Večna Pot 113, 1000 Ljubljana, Slovenia; 6 The Novo Nordisk Foundation Center for Protein Research, University of Copenhagen, Blegdamsvej 3B, 2200 Copenhagen N, Denmark; 7 National Museum of Natural History, Smithsonian Institution, Washington, DC 20560 USA; 8 Nunatta Katersugaasivia Allagaateqarfialu, Greenland National Museum & Archives, Hans Egedesvej 8, Boks 145, 3900 Nuuk, Greenland; 9 The National Museum of Denmark, Modern History and World Cultures, Frederiksholms Kanal 12, 1220 Copenhagen K, Denmark

**Keywords:** Mass spectrometry, Proteomic analysis

## Abstract

Until recently, the identification of the species of origin for skin and fur materials used in the production of archaeological clothing has been based on the analysis of macro- and microscopic morphological features and on the traditional knowledge of Indigenous groups. This approach, however, is not always applicable due to the deterioration of the archaeological objects. Paleoproteomics was used as an alternative approach to identify the species of origin of fifteen samples of various tissues from approximately 600-year-old garments found in Nuulliit, northern Greenland. Proteomics revealed that a limited group of marine and terrestrial mammals were used for clothing production. The results obtained from the analysis of multiple types of clothing and elements, such as sinew thread and gut skin, suggest that their applications were based on their properties. When conclusive assignment of a sample to a species via proteomics was not possible, the observation by transmitted light microscopy of feather and hair micromorphology, if not affected by diagenesis, was used to improve the identification. The proteomic characterization of animal materials used for clothing production in the Nuulliit archaeological context provides an insight into the practical knowledge and the strategies adopted by the local Indigenous community to exploit natural resources.

## Introduction

Clothing is extremely important to human adaptation and survival because it “conserves heat, eliminates humidity, controls temperature, [and] prevents ingress of wind and water”^1^, especially in high latitude environments. Before the introduction of weaving, clothing was often produced from protein-based biomaterials, such as animal skins and furs, that naturally had the same protective function in animals^2^. Since the nineteenth century, the species of origin of archaeological skin objects have been tentatively identified by Indigenous experts, and/or by comparing their appearance with known materials and similar museum objects using both macro- and microscopic observation^3–5^. These approaches, however, rely on the expertise of a few specialists in the field. Material classifications can be challenged when the garments are heavily altered, or when no relevant comparative materials are available. Additionally, hollow organ connective tissue and skin tissues from different species can have very similar morphology and appearance^6^, and hair shape can even vary within the same species, in relation to the age, sex, nutrition, health state, and sampled area of the hide^7^. Therefore, the reliability and reproducibility of macro- and microscopic visual interpretations of the biological origin of skin and hair objects can sometimes be insufficient to achieve species-level identifications. In some cases, these visual methods have been shown to lead to inconsistent conclusions when compared to molecular methods such as mass spectrometry (MS)-based ancient protein sequencing^8^.

The analysis of ancient biomolecules, i.e. DNA and proteins, can overcome some of the limits of traditional identifications, providing confident and accurate species attribution even for very ancient and damaged objects. However, both of these analyses require the collection and destruction of a (micro) sample to perform the analysis, and their results can still be affected by diagenesis and modern contamination. The recovery of ancient DNA is also prone to failure if the objects have been buried in acidic environments, such as peat bogs, severely hampering its preservation^8^, and ancient protein analysis can be limited by the gaps in publicly available protein reference databases. Despite these limits, over the past few years paleoproteomics has gained more and more relevance^9^. Specifically, it has been used to identify clothing material from several contexts, such as Bronze Age sites along the Silk Road^10^, Viking Age Denmark^11^, and Pre-Columbian Peru^12^. The garment proteins identified are mainly keratins from fur, or collagen from skin^13^, and, less frequently, hollow organ sub-mucosa. In the literature, the terminology currently used to refer to the latter class of materials is “gut skin” or “intestinal skin”. As it is currently impossible to determine which hollow organ is the exact source of the material used to produce the investigated garments, the term “gut skin” is adopted hereafter to conform with the terminology used so far^14–16^.

Among protein-based techniques, tandem MS (MS/MS) provides higher accuracy in the identification of the species, thanks to the possibility to confidently characterize the amino acid sequence of the detected peptides^17,18^. Recent applications to archaeological textiles have shown the power of protein sequencing to discriminate keratin sequences even when originating from taxonomically close species^10,12,19,20^. Therefore, we used paleoproteomics to identify the species of origin of eight skin or gut skin clothing elements, Table 1 (further images in Figs. S1–S10), from the site of Nuulliit (previously Nûgdlît, Lat: 76° 33′ 0ʺ N, Long: 68° 48′ 0ʺ W) in northern Greenland, dated to the fourteenth century AD and including some of the oldest gut skin parkas (Greenlandic: ikiaq/annoraaq) found in the Arctic (samples A, B, and C in Table 1)^21^. The site was investigated in 1947 by the Danish archeologist Erik Holtved and an Inughuit team from northern Greenland traveling with him. The archaeological site provided evidence of the prehistoric hunting habits of Inuit people in Greenland, including the wide availability of materials from local species such as caribou, seals, and whales^22^, and the use of skins from land and sea mammals, with or without fur, and feathered bird skins^1^. Waterproof garments were produced by stitching together long strips of connective tissue retrieved from hollow organs^1^. Finally, the Inuit used gut skin and tendons as sewing thread to produce waterproof stitching^1^. Learning about the choice of materials used to produce clothing and, if possible, associating the use of materials from different species with practical, symbolic or social/aesthetic functions, are of great importance to better understand the culture and values of the immigrant Inuit community in the Nuulliit area. The aim of this work is thus to understand whether materials originating from certain animal species were deliberately used to preferentially, or exclusively, produce specific garment elements such as fabrics (gut skin, de-haired skin, dermal skin, fur skin, or bird skin) and/or sewing thread (sinew or gut skin). Furthermore, the species identification could help in the reconstruction of interactions of different peoples and groups, such as migration and trade. Although gut skin has been investigated with proteomics before^23^, this work describes, to the best of our knowledge, the first application of tandem MS-based proteomics for the identification of the species of origin of archaeological gut skin clothing. Two samples from a parka, dated to the mid twentieth century, here referred to as “historical parka”, were used to test the protocol, previously developed to investigate archaeological and artistic materials^24,25^, on gut skin and sinew thread materials. Regardless of origin, the sewing thread is hereafter named ‘sinew’. Based on this preliminary test, the sample preparation workflow was modified introducing a cleaning step before protein extraction, to remove lipidic conservation substances and pesticides, known to have been used on archaeological specimens preserved at the National Museum of Denmark^26^. Once it was assessed that the optimized protocol could successfully identify the species of origin of gut skin and sinew thread, fifteen samples were removed and analyzed from eight archaeological clothing specimens from the Nuulliit site (Table 1).

**Table 1:** Survey of samples.

	Specimen code	Location	Holtved 1954 (in short) (47)	Description, condition 2022	Sampled portion	Analyses
	A	Nuulliit, house 28	Gut skin jacket. Body with vertical strips Hood with horizontal strips. Trimmed with depilated seal skin. Fragmented	Parka: of gut skin with hood, sleeves, curved lower borders. Hood opening, sleeves and lower border trimmed with de-haired seal skin. Fragmented condition, contaminated with pesticides. Back height ca. 140 cm, fathom width ca. 136 cm Gut skin: dark brown, partly translucent, greasy. 4–8 cm wide guts, ca. 0.5 mm thick. Brittle condition. Shrinkage temperature, main interval: 41–48 °C Borders: de-haired seal skin trim ca. 1 cm wide Sewing (sinew): slightly twinned thread, diameter ca. 1 mm. Overcast stitch	Gut skin Thread	MSPS MSPS
	B	Nuulliit, house 28	Gut skin jacket. Body with vertical strips Hood with horizontal strips. Hood with vertical strips Fragmented	Parka: of gut skin with hood, remnants of sleeves; curved lower borders. Fragmented condition, contaminated with pesticides. Back height ca. 95 cm Gut skin: brown, opaque. Cohesive condition. Ca. 14 cm wide guts, ca. 1 mm thick. Shrinkage temperature, main interval: 40–53 °C Sewing (sinew): slightly twinned thread, diameter ca. 1 mm. Overcast stitch	Gut skin Thread	MSPS MSPS
	C	Nuulliit, house 28	Gut skin jacket. Body with vertical strips Hood with horizontal strips. Fragmented	Parka: of gut skin with hood, remnants of sleeves, curved lower borders. Fragmented condition, contaminated with pesticides. Back height ca. 108 cm Gut skin: brown, opaque. Brittle, but cohesive condition. Ca. 8–11 cm wide guts, ca. 1 mm thick. Shrinkage temperature, main interval: 42–53 °C Sewing (sinew): slightly twinned thread, diameter ca. 1 mm. Overcast stitch	Gut skin Thread	MSPS MSPS
	D	?	Not mentioned	Parka (?): of gut skin, back (?). Fragment, contaminated with pesticides. Height ca. 78 cm Gut skin: brown, contaminated with pesticides. Brittle, but cohesive condition. Ca. 8 cm wide guts, ca. 1 mm thick Sewing (sinew): slightly twisted thread, diameter ca. 1 mm	Gut skin	MSPS
	E	Nuuliit, house 22	Fragment from gut skin jacket, bordered with hairy seal skin	Parka (?): of gut skin. Two fragments contaminated with pesticides. Heights ca. 40 cm and 16 cm Gut skin: brown, partly translucent. Brittle, not cohesive condition. Shrinkage temperature, main interval: 45–54° C. Ca. 8 cm wide guts, ca. 0,5 mm thick Border: fur skin ca. 1 cm wide Sewing (sinew): slightly twisted thread, diameter ca. 1 mm. Overcast stitch	Gut skin Thread	MSPS Hair TLM MSPS
	F	Nuulliit, house 28	Top part of woman’s boot or stocking of dog skin with a strip for fastening. Fragment	Footwear top part of fur skin with trimming of the same fur. Fragmented condition, contaminated with pesticides. Length ca. 45 cm Fur skin, shaft: skin ca. 2 mm thick Trimming: fur skin strips, ca. 2 cm wide Sewing (sinew): slightly twisted thread, diameter ca. 2 mm. Overcast stitch	Dermal skin fur	MSPS MSPS Hair TLM
	G	Nuulliit, house 28	Hood of bird skin. Dog fur border	Hood of bird skin with fur trim. Loose strap 52 cm long. Fragmented condition, contaminated with pesticides. Height ca. 39 cm Bird skin: with plumage Trimming: fur skin, ca. 1 cm wide Sewing (sinew): slightly twisted thread, diameter ca. 1 mm. Overcast stitch	Bird skin Fur	MSPS Feather microscopy MSPS Hair TLM
	H	Nuulliit, house 28	Trouser leg? of bird skin with dog fur border. Fragmented	Parka sleeve (?) of bird skin with fur trim. Fragmented condition, contaminated with pesticides. Height ca. 55 cm Bird skin: with plumage Trimming: fur skin, ca. 1 cm wide Sewing (sinew): slightly twisted thread, diameter ca. 1 mm. Overcast stitch	Dermal skin Fur	MSPS Feather microscopy MSPS MSPS Hair TLM
Historical reference						
V b	R	Greenland; collected by Jens Rosing mid-twentieth century	–	Parka of gut skin with fragmented sleeve Height 63 cm, length 75 cm	Gut skin Thread	MSPS MSPS

## Results

Numerous proteins and peptides were identified in all the analyzed samples. The identified peptides originated predominantly from collagens in the gut skin and thread samples, and from keratins in the fur samples. The proteomic analysis of the fifteen archaeological and two historical samples allowed for the identification of marine species such as seals, walrus, and whale, and terrestrial species such as fox, dog, and polar bear. The complete list of proteins confidently identified for each sample is provided in Table 2. Information about total recovery of proteins and peptides in the case of complete species proteome availability is provided in Table 3. Details about protein and diagnostic peptide identification are in Supplementary Tables 1 and 2. The results regarding historical and archaeological garments are discussed below based on the material type analyzed: thread, fur, gut skin, and other types of skin.

**Table 2:** Type of proteins and species identification for each sample analyzed.

Specimen code	Source	Species	Main proteins	Notes
R	Gut skin	*Phoca vitulina* (harbour seal)	Collagens	Possibly *Pusa hispida* (ringed seal)
	Sinew thread	*Bos/Muntiacus /Odocoileus*	Collagens	Possibly *Rangifer tarandus* (caribou) or *Ovibos moschatus* (Musk ox)
A	Gut skin	*Phoca vitulina* (harbor seal)	Collagens	Possibly *Pusa hispida* (ringed seal)
	Sinew thread	*Delphinapterus leucas* (Beluga)	Collagens	
B	Gut skin	*Odobenus rosmarus divergens* (Pacific walrus)	Collagens	Possibly *Odobenus rosmarus rosmarus* (Atlantic walrus)
	Sinew thread	*Balaenoptera acutorostrata scammoni* (Minke whale)	Collagens	Possibly *Balaena mysticetus* (bowhead whale)
C	Gut skin	*Odobenus rosmarus divergens* (Pacific walrus)	Collagens	Possibly *Odobenus rosmarus rosmarus* (Atlantic walrus)
	Sinew thread	*Balaenoptera acutorostrata scammoni* (Minke whale)	Collagens	Possibly *Balaena mysticetus* (bowhead whale)
D	Gut skin	*Odobenus rosmarus divergens* (Pacific walrus)	Collagens	Possibly *Odobenus rosmarus rosmarus* (Atlantic walrus)
E	Gut skin	*Odobenus rosmarus divergens* (Pacific walrus)	Collagens, muscle proteins	Possibly *Odobenus rosmarus rosmarus* (Atlantic walrus)
	Sinew thread	*Odobenus rosmarus divergens* (Pacific walrus)	Collagens, muscle proteins	Possibly *Odobenus rosmarus rosmarus* (Atlantic walrus)
F	Dermal skin	*Canis lupus familiaris*	Collagens	Possibly *Canis lupus*
	Fur	*Canis lupus familiaris*	Keratins	Possibly *Canis lupus*
G	Bird skin	Aves	Collagens	Duck or other bird species
	Fur	*Ursus maritimus*	Keratins, skin proteins	
H	Dermal skin	*Vulpes lagopus*	Collagens	
	Fur	*Vulpes lagopus*	Keratins, skin proteins

**Table 3:** Total recovery of proteins and peptides for samples in which complete species proteome was available.

Sample	Identified proteins	Total peptides	MS/MS spectra
R gut skin	23	279	771
A gut skin	6	167	714
A sinew thread	4	147	599
B gut skin	11	285	1592
B sinew thread	8	174	1361
C gut skin	7	141	416
C sinew thread	6	154	906
D gut skin	6	169	563
E gut skin	22	465	2381
E sinew thread	19	414	2094
F dermal skin	12	281	1824
F fur	40	457	6824
G fur	45	457	4113
H dermal skin	23	352	1858
H fur	39	413	5115

### Protein damage

Deamidation is a protein modification occurring on asparagine (Asn, N) and glutamine (Gln, Q) residues that is usually, but not unanimously^27^, considered an indicator of protein damage accumulating over extended time intervals^28–30^. In all the samples, the deamidation percent ranged from 6 to 43%, and from 4 to 26% for Asn and Gln respectively. For almost all specimens, fur and thread were on average less deamidated than dermal and gut skin (Fig. S11). Gut skin and thread showed comparable deamidation in sample E, and for sample A the thread was slightly more deamidated than the skin. In general, the glutamine deamidation rate of the dermal skin samples was from 1.5 to 3 times higher than that of the historical Parka R, in accordance with their greater antiquity. Gut skin jacket E presented comparable values to the historical Parka R for Gln deamidation (around 9%) but higher values for Asn residues (around 34% in E, around 26% in R). Considering that Gln deamidation is more representative of aging processes, this specimen’s proteins were probably better preserved because of environmental factors^27^. In general, the obtained results indicate that the proteins from the analyzed samples were probably older than those from the historical Parka R, and thus can be considered endogenous and authentically ancient.

### Historical garment

The historical gut skin and thread samples (Parka R) were analyzed first to verify the efficacy of the modified proteomic protocol. The additional steps for the elimination of pesticides and fatty materials did not hinder the analysis and allowed the identification of numerous proteins. In particular, the proteomic analysis of the gut skin sample revealed that this material originated from seal. Marker peptides indicate harbor seal (*Phoca vitulina*) as the species, although the protein sequences of other relevant seal species known to live in the area surrounding the Nuulliit site, such as ringed seal (*Pusa hispida*) and bearded seal (*Erignathus barbatus*)^31^, are not available in public databases of protein sequences and cannot be used for comparison. Many collagen sequences, as well as other proteins such as serum albumin and myosin-11, were identified (Table 2). The biological source of the sinew of Parka R was a species in the Pecora infraorder (even-toed hoofed mammals). Two peptides specific for Pecora species were identified, but a more diagnostic peptide allowed the identification to be narrowed down to bovine (*Bos*), muntjacs (*Muntiacus*) or deer (*Odocoileus*) species. Collagen databases are still limited for wild species, so matches with the protein sequences of other species, not publically available, cannot be excluded. Species in the Pecora infraorder and endemic to Greenland were considered, in particular caribou (*Rangifer tarandus*) and musk ox (*Ovibos moschatus*), which live in this area. Indeed, it is known that in the past Inuit used dorsal tendons from caribou as sewing thread, a practice which continues to this day^1^. Musk ox collagen sequences are not available, and only a partial sequence of collagen 1 is available for caribou^32^. One of the recovered peptides (IGQPGAVGPAGIR) would militate against the identification as caribou, as it does not match the same position in the partial caribou sequence (AGQPGAVGPAGIR). Musk ox is thus likely to be the tentatively identified source species of the material, although comparative musk ox collagen sequences and more complete caribou sequences should be used to confirm this result.

### Archaeological garments

#### Sinew thread

The sinew thread used in all the archaeological garments investigated were produced from marine mammals. In detail, the sinew thread of Gut skin jacket A was produced from beluga whale (*Delphinapterus leucas*). A diagnostic peptide allowed the discrimination between beluga and narwhal (*Monodon monoceros,* see alignment and spectrum Fig. S12). Protein sequences matching those from minke whale (*Balaenoptera acutorostrata scammoni*) were identified in the sinew thread samples from both Gut skin jackets B and C. While minke whale is one of the whale species living in Greenlandic waters, it is closely related to bowhead whale (*Balaena mysticetus*), whose collagen sequences are not available, thus the latter cannot be excluded as an identification for these samples. For Gut skin jacket E, protein sequences matching those from walrus (*Odobenus rosmarus*) were identified. In this sinew thread sample, in addition to collagens, walrus-specific proteins such as serum albumin and hemoglobin were also identified. The identified protein sequences matched those of the Pacific walrus subspecies (*O. r. divergens*). At the same time, the use of Atlantic walrus (*O. r. rosmarus*), for which protein sequences are not available, cannot be confidently excluded at this stage.

#### Gut skin

In all the gut skin samples, proteins from pinniped species were identified. In particular, the gut skin of Gut skin jacket A, similarly to Parka R, contained collagen from harbor seal (or possibly ringed seal, *Pusa hispida*, see Discussion). For all the other gut skin samples (Gut skin jackets B, C, and E, and Gut skin parka D), walrus (*Odobenus rosmarus*) was identified as the source of the materials. As already mentioned, although the peptides are specific to Pacific walrus, Atlantic walrus cannot be excluded. In Gut skin jacket E, the gut skin presented not only walrus collagen proteins such as collagen alpha-1(I), and collagen alpha-2(I), but also serum albumin, hemoglobin, and other non-collagenous proteins, including antithrombin-III, apolipoprotein A-I, Ig lambda chain V-I region BL2, and bone marrow proteoglycan.

#### Other types of skin

Dermal skin samples from two specimens, F and H, contained Canidae proteins. In particular, the dermal skin sampled from F presented peptides specific for the species *Canis lupus*. However, it was not possible to distinguish between two subspecies: domestic dog (*Canis lupus familiaris)* and wolf (*Canis lupus lupus)*. As expected, collagens were the main identified proteins. In Trouser leg H, species-specific collagen peptides from Arctic fox (*Vulpes lagopus*) were identified*.* In this case it was possible to exclude red fox (*Vulpes vulpes*) due to sequence availability of both species. As an example, a sequence alignment and the corresponding spectrum of one of the diagnostic peptides of Arctic fox compared to red fox is reported in Fig. S13. In the skin sample of Trouser leg H, hemoglobins, myosin, and other proteins were identified in addition to collagens (Table 2 and SI). The particular manufacturing of this specimen, made of feathered bird skin with a fur ribbon on the edge, allowed for the microscopy analysis of both the feathers of the main body and the hairs of the fur ribbon. The microscopic identification of Arctic fox from the hair (Fig. S14), matches the results of the proteomic analysis, which was performed on samples removed exclusively from the skin and fur of the outer ribbon. In the case of Hood G, the appearance suggested the use of bird skin. Indeed, bird proteins were identified by proteomic analysis, although the species could not be established, as peptide sequences indicating multiple bird species were identified: mallard duck (*Anas platryhynchos*), black-tailed godwit (*Limosa limosa*) and great cormorant (*Phalacrocorax carbo*). All these species are geographically plausible. It is unlikely that collagen proteins from more than one species would be present in this context, since the object was presumably made with one layer of skin. However, it cannot be excluded that the whole object was originally made of bird skin from multiple species, as previously observed in a bird skin parka made from at least five different bird species from the Qilakitsoq site in western Greenland (dated to AD 1475^33^). Thus, the presence of proteins from different bird species could be due to cross-contamination from different portions of the same object. Nonetheless, this result more probably indicates that the protein sequences of the species of origin for Hood G are not present in publicly available databases, leading to the preliminary assignment of the identified set of peptides to more than one species at the same time. The microscopy analysis of feathers removed from this specimen suggests their origin from a species of the Alcidae family, such as auks, murres, and puffins (Fig. S15). Relevant protein sequences for species in this family are not available, therefore the identification of the exact species cannot be achieved with either method at this time.

#### Fur

The fur samples of the two specimens F and H contained Canidae proteins. The fur sampled from Boot/Stocking F presented wolf*-*specific peptides, while the fur sampled from Trouser leg H contained keratin peptides specifically from Arctic fox. Microscopy performed on hair from the Boot/Stocking F (SI) may suggest that the fur was from a domestic dog. However, with longitudinal and cross-sectional mounting examination by microscope, hairs from dog and wolf have a relatively similar appearance and size, making subspecies identification still questionable^34^, see Fig. S16. In the proteomic analysis, keratins were the main components identified, although collagen alpha-2(I) and some other proteins were also found (Table 2). Species-specific keratin and desmoplakin sequences from polar bear (*Ursus maritimus*) were identified for the fur of Hood G. In this sample other proteins such as 17-beta-hydroxysteroid dehydrogenase 14 and methanethiol oxidase were also identified. Polar bear was also identified through microscopy analysis of the hair, see Fig. S17.

## Discussion

The results obtained from the paleoproteomic analysis of eight garments from the Nuulliit archaeological site confirmed that materials from different animal species were used in the same object. This was observed particularly in different elements of the garment, which are typically sewn together in culturally significant patterns^1,35,36^. The sampling was limited to no more than one sample per garment element type, thus these results are limited to the elements investigated. Future research should expand this work to achieve a broader characterization of the use of different species in relation to the sewing patterns (Figs. S1–S10 and S20). The results obtained in this study show that a limited variety of species was used, often for different purposes, and probably carefully chosen based on specific mechanical and cultural characteristics. In our set of objects, the proteins we identified showed that threads were made from whale and walrus, gut skins from seal and walrus, other types of skins from dog, fox and bird species, and fur from dog, fox and polar bear. An interesting trend was found in the types of animals used in a single piece. Materials from marine and terrestrial animals do not appear to have been used together in the same piece of clothing, for example by using the gut skin of a marine mammal and the sinew thread of a terrestrial animal, in any of the archaeological specimens. This was instead observed in the historical sample, where proteins matching seals were identified in the gut skin and proteins from terrestrial species were identified in the thread, possibly reflecting the changing modes of production and/or cultural values in the communities of this region. Among the nearby Canadian Inuit, there were strict rules and taboos against skin clothing production, and the mixing of raw materials of terrestrial and marine origin, including caribou, seals and walruses^37^.

Several marine species were identified throughout the sample set, in particular as the source of the gut skin. Gut skin parkas were produced in Greenland at least until the nineteenth-twentieth century^38–40^. In the late eighteenth century in western Greenland, gut skin from several seal species was used mainly for men’s clothing, tent curtains, and windows^41^. In the twentieth century, Inuit in Alaska still used gut skin from bearded seals and walrus for sea hunting parkas and ceremonial clothing^1^ and both men and women used gut skin parkas^35^. In the samples analyzed here, seal was identified in only one gut skin sample (Gut skin jacket A). In the same specimen, proteins from Cetacea were identified in the thread. Four out of six pieces of clothing (B, C, D, and E) were instead made using walrus gut skin, and walrus, beluga, or whale thread. The predominance of walrus in these garments reflects its abundance in the zooarchaeological remains from Nuulliit^42^, where only the remains of bowhead whales are more abundant. The only case in which the thread was made from walrus (E) also showed a more distinct proteomic profile. In all other specimens, the gut skin samples only contained collagens, whereas in Gut skin jacket E non-collagenous proteins including for example antithrombin-III and apolipoprotein A-I, were also identified. The detection of muscle proteins, and in both the gut skin and sinew thread of this specimen, might reflect that different techniques were used to prepare this specific garment. Different protocols are known to have been used in different geographical and historical contexts, such as (i) removing the inner and outer layers of the gut skin and soaking in urine, as done in the twentieth century by Alaskan Iñupiat^43^; or (ii) pressing the gut skin between two fingers, soaking in blubber for a few days, and chewing the gut skin, as done in western Greenland in the eighteenth century^41^. However, the degradation profile of these proteins is unknown, and thus differences in the proteomic profiles might be related to the preservation status of the garments rather than to the preparation technique. As for the sinew thread samples, a species of the Pecora infraorder was identified as the source for the historical specimen. Tendons from whales, narwhal, beluga whales, waterfowl, arctic fox, polar bear, and musk ox were instead used in the archaeological specimens^1^. Literature sources report that the most suitable sinew thread was derived from caribou tendons^44^, a species that was not identified here.

Terrestrial species were identified in specimens F, G, and H, in agreement with the finding of skeletal remains of dog, Arctic fox, and polar bear, among other terrestrial mammals, at the Nuulliit site^31^. In the case of Boot/Stocking F, proteomic analysis on both the dermal skin and the fur suggested either dog or wolf as the source animal, but could not distinguish between the two. This is due to the limited information about wolf protein sequences and the taxonomical proximity of the two subspecies. Through microscopy analysis, the structure of the hairs suggested that the species of origin was domestic dog.

All over the Arctic, up to the beginning of the twentieth century, the Inuit people used bird skin to produce clothing, often preferring diving waterfowl because of their tougher skin compared to non-diving birds^1^. For Hood G, several species-specific peptides were identified, albeit for three different bird species. This result suggests that the protein sequences of the actual species of origin are not present in public databases, but this species is probably closely related to the three whose peptides were found: mallard duck, black-tailed godwit, and great cormorant. For both specimens G and H, microscopy techniques were used to try and identify the species based on the characteristics of the plumulaceous feather barbs^45^. In both samples, the species of origin was identified as an Alcid, probably an auk, a murre, or a puffin. Indeed, the skeletal remains of thick-billed murre were found to be the most common among bird species at the Nuulliit excavation site, followed closely by little auk^31^. Protein sequences of this family of birds are lacking in public databases; however, mallard and great cormorant are taxonomically distant from Alcids, whereas black-tailed godwit is also part of the order of the Charadriiformes. Therefore, the species of origin of the feathers for Hood G probably belongs to the Alcid family and is closely related to black-tailed godwit. For specimens G and H, the appearance of the skin suggested that the material was bird, as evidenced by the typical feather follicle pattern, visible to the naked eye, and the presence of feathers. However, for Trouser leg H, bird proteins were not identified in the proteomic analysis. This is probably because the sample was removed from the edge of the object, where a fur ribbon is applied. This was traditionally done with the skin and/or fur of Arctic fox, dog, polar bear, and seal, in order to reinforce clothing made of bird skin^1,35,40^. The collected sample comprised skin and fur and was therefore divided to try and determine the species of origin of the two tissues separately. Only proteins from Arctic fox were identified in both the dermal skin and the fur samples from this specimen, indicating that only the dermal skin attached to the fur was actually sampled. The risk of the species identifications being due to the presence of conservation materials should also be considered. If animal glue had been used as a consolidant, for example, collagens from the glue would have probably been identified. However, such treatments would likely have occurred once the objects were brought to Europe. Thus, the identification of species absent or very uncommon in Europe, and on the other hand known to be present in Greenland, supports the authenticity of the results. Furthermore, the differences in terms of protein composition (keratins in the fur portion of the sample and collagens in the skin portion) suggest the presence of two different tissues rather than two types of glue.

In this work, the species of origin was identified for most of the samples studied, although the results highlighted the limits of available protein sequences in public databases. This is a known problem for wild species identification in archaeological materials^11^. For example, the proteomic results suggested that a species of seal was the source of the gut skin for both the more recent Parka R, and Gut skin jacket A. This was based on the identification of peptides from harbor seal. However, it has to be considered that relevant protein sequences from bearded seal and ringed seal, both previously reported in the zooarchaeological remains from Nuulliit^42^, are not available in current protein sequence databases. The sequences cannot therefore distinguish between harbor, bearded, and ringed seal. Nonetheless, harbor and ringed seal are closely related. Based on the identification of peptides distinguishing harbor seal from Weddel seal species, which is present in databases and closely related to the bearded seal (Fig. S18), the species of origin is probably not the latter, but more likely either harbor or ringed seal. Literature sources, previous studies and other analytical techniques might compliment proteomic results when the lack of protein sequences makes it challenging, or even impossible to confidently claim the origin of the material. However, this was not the case for these samples, since the use of ringed seal is also known for this site^31,42^.

The same reference sequence limitations were faced for the identification of gut skin and sinew thread produced from walrus, with the Pacific and Atlantic subspecies being at the moment indistinguishable, and for thread assigned to minke whale, which might instead derive from bowhead whales. The identification of Pacific or Atlantic walrus would be archaeologically relevant due to the assumed migration pattern of the community from Alaska to Greenland^46^. Thus, further investigations to obtain the protein sequences of the Atlantic walrus and discriminate the two subspecies should be envisaged.

Despite the limitations imposed by the lack of protein sequences for several wild Arctic animals, this work proves the value of this technique for the identification of the species of origin of archaeological materials, such as gut skin and sinew, for which microscopic morphological features are not always sufficient. The public availability of the raw mass spectrometry data generated in this study, see Data deposition note, enables their future re-analysis, once richer databases for the species of interest are made publicly available. This will allow further refining of the interpretations presented here with no need to sacrifice and re-analyse more irreplaceable archaeological material. The novel implementation of a cleaning step before protein extraction minimized the interference of pesticides and other additives used in conservation treatments on the proteomic analysis.

This work is part of a larger interdisciplinary study investigating the relationships among social roles, animal skin types, skin clothing production and design, and the circumpolar people’s way of life and interactions. For the first time, this study aimed at the identification of the animal species of origin of all the elements (primary material, edging, and sewing thread) used to produce gut-skin garments connected to an immigrant Thule whaling community in the fourteenth century. It also brings light to previously unknown patterns in animal use to produce garments in this cultural and geographical context, thus far only investigated by visual examination or by comparison with the zooarchaeological record retrieved in the archaeological site.

## Materials and methods

### Materials

**Figure 1:**
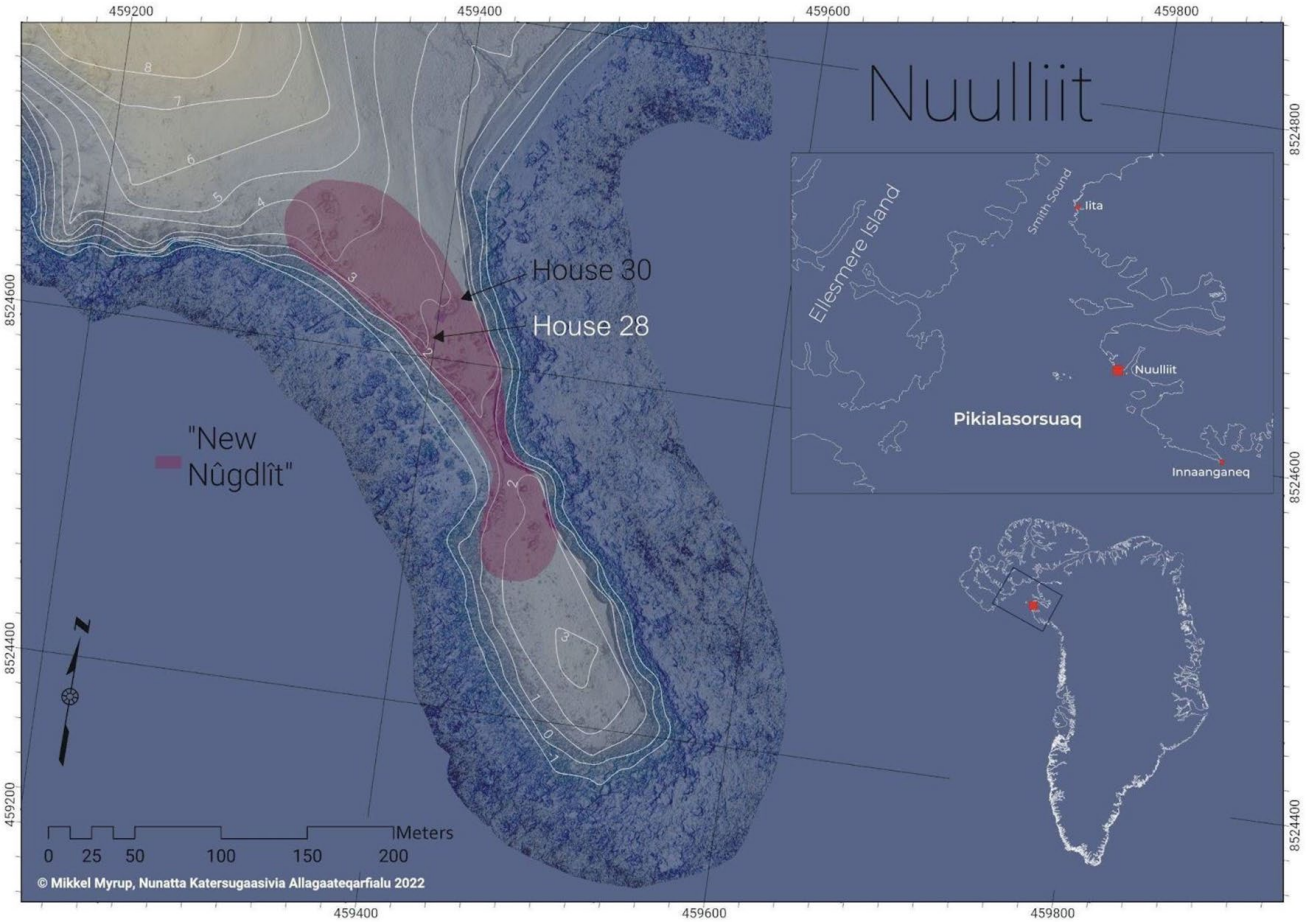
Map of Thule area and survey plan of ruin site.

No less than 51 winter houses were found in the site of Nuulliit (previously Nûgdlît, Lat: 76° 33′ 0"N, Long: 68° 48′ 0" W) in northern Greenland. Most of these are associated with the early Inuit whaling community (Thule Culture), which spread from Canada to Greenland across the Nares Strait. Originating in Alaska, pioneering groups of the Thule Culture probably reached Greenland as early as the twelfth century, but a more permanent settlement in Greenland as represented by the Nuulliit site was first established in the early fourteenth century^31,47^. Well-preserved portions of clothing made of various animal gut skin and furred and feathered skins, as well as hunting equipment, utensils, household tools, toys, and other artifacts were discovered in permanently frozen layers in some of the ruins. Six of the specimens sampled in this study were discovered in the ruins of house 28 (Fig. 1). In most cases, two samples were taken from the same object to determine the species of the main material (dermal or gut skin) and of the thread used. Samples from fur were also collected where clearly visible (specimens F–H).

Upon formal request, the Greenland National Museum allowed sampling and registration of the Nuullit skin collection. Fifteen samples from eight examples of clothing from the Nuulliit archaeological site (Table 2), belonging to the National Museum of Greenland and currently located at the National Museum of Denmark, were studied using proteomic analysis. Samples of < 1 g were removed from the object, in particular on the edges of the thread and/or fabric, or where damage had already occurred and small portions could be removed without impacting the integrity of the specimens. Sub-samples were used for proteomic analysis, whereas the biggest part of the original samples were preserved for future studies. Three parkas of gut skin (specimens A, B, and C—Table 1) were excavated from the site. The garments all had similar construction: the body and sleeves were sewn from horizontal strips, and the hood back neck had vertical strips in one parka, while horizontal in the other two. The hood had inserted double hood roots at the front and back. The parkas’ lower border at the front and back were curved. In house ruin 22, fragments from a possible parka with a fur skin trim (Gut skin jacket E) were also found^47^. In addition, a large cohesive parka fragment (specimen D), ascribed to the Nuulliit finds but not recorded in the literature, was included in this study. In addition to the gut skin parkas, three bird skin garments were present: a fragmented part of a parka with a fur trim preserved on the lower part; a loose hood with fur trim and loose strap (Hood G); and what is thought to be either a leg for a child’s trousers or a sleeve from a parka with a fur trim (Trouser leg H)^47^. Two fragmented garments of fur skin were found: (1) an upper shaft of a piece of footwear made of fur skin (not included in this study), and (2) the upper trimming of a piece of footwear made of fur skin (Boot/Stocking F)^47^. A 100-year-old clothing fragment made of gut skin and sinew thread, here referred to as “historical parka”, was used as a reference sample (Parka R). The specimen belongs to the former Greenlandic Landsmuseum (catalog number 704), and it was left to the Danish National Museum as test material in 1977 by the former director Jens Rosing. This sample was analyzed first to test the feasibility of the extraction method before sampling from the archaeological samples.

### Methods

#### Feather and hair microscopy

Samples were prepared following Dove and Peurach^45^ A Leica DM4 M with transmitted light (TL) (100x, 200x, 400x, and 500 × magnification) was used for microscopy of the hair. Feather material was examined microscopically using a Leica^©^ DM750 (Leica Microsystems, Wetzlar, Germany) comparison light microscope at 50x, 100x, 200x, and 400x (power). Photomicrographs were taken with a Leica^©^ DFC290 HD camera (Leica Microsystems, Wetzlar, Germany). Further information in SI.

### Proteomic and mass spectrometry analysis

Two micro-samples of a historical Greenlandic garment (Parka R), dating to the mid twentieth century, were removed from the gut skin and the sinew thread, respectively. The samples were analyzed prior to the archaeological samples to assess the proteomic approach and compare the aging process. Furthermore, a laboratory blank was processed in parallel with the samples and analyzed in the same way. Information about the storage of the historical reference is unknown. Therefore, it cannot be ruled out that the garment was treated with biocides. It is however known that the archaeological samples were treated with biocides and lipids such as neatsfoot oil and castor oil from conservation treatments^26,48^. Other naturally occurring lipids could also affect the analysis. Thus, a protocol for the elimination of compounds interfering with protein extraction was tested on the historical parka, and then applied to the archaeological samples. A mixture of hexane/acetone (1:1) was added to each sample, in the volume necessary to submerge the sample completely (< 500 uL), heated at 50 °C and agitated at 700 rpm for 5 min. The samples were then cooled down and centrifuged. The supernatant was removed, and the steps were performed at least three times. This wash procedure was extended for the samples in which the supernatant was still very colored after the third wash. The samples were then dried, and protein residues were extracted from the samples using a lysis buffer as in Mackie et al.^25^. The obtained tryptic peptides mixtures were separated on a 15 cm column (75 μm inner diameter) in-house laser pulled and packed with 1.9 μm C18 beads (Dr. Maisch, Germany) on an EASY-nLC 1200 (Proxeon, Odense, Denmark) connected to a Q-Exactive HF-X (Parka R) or Exploris 480 (archaeological samples) The instruments were operated in data dependent top 10 mode. Further details are available in SI.

### Database search and proteins identification

The MS/MS spectra were identified with the MaxQuant software 1.6.3.4^49^, matching them against two reference databases, containing (i) publicly available sequences of collagen 1 and collagen 2 of Mammalia for dermal skin, gut and sinew thread samples, and (ii) publicly available sequences of keratins and keratin-associated proteins for fur samples. In the case of sample G, presenting a bird skin appearance, the search was performed against a database containing all the publicly available sequences of collagen of Aves. This search allowed a preliminary assignment of the samples to a biological species, or to a restricted group of taxa. The matches were against fully tryptic peptide sequences, with no taxonomic restriction. The following were set as variable modifications: oxidation of methionine, deamidation of asparagine and glutamine, conversion of N-terminal glutamine to pyroglutamic acid, conversion of N-terminal glutamic to pyroglutamic acid, and hydroxyproline. Carbamidomethylation was set as a fixed modification. The minimum peptide length was set to 7 amino acid residues, with up to two missed cleavages. The false discovery rate (FDR) was set to 0.01, whereas the minimum score for both unmodified and modified peptides was set to 40. The error tolerance was set to 5 ppm for the precursor and to 20 ppm for the fragment ions. Contaminant proteins were assessed using the contamination.fasta provided by MaxQuant (http://www.coxdocs.org/doku.php?id=maxquant:startdownloads.htm). Peptides assigned by the software to contaminant proteins were filtered out and not considered further. Peptides were considered diagnostic when, after BLAST search against the entire nrNCBI protein database, they were assigned to a single species, or to a limited number of species among which only one can be considered plausible, based on the nature, geographic origin, and dating of the sample. Once the species was identified, the MS/MS spectra were searched against a database containing the complete proteome of the species. The damage over time was evaluated by calculating the percentage of deamidation, as described in Mackie et al.^25^.

### Supplementary Information

Supplementary Information.

Supplementary Tables.

## Data Availability

The mass spectrometry proteomics data have been deposited to the ProteomeXchange Consortium via MassIVE (https://massive.ucsd.edu/) with the dataset identifier MSV000091145.
