# Supplementary material for: Paleoproteomic identification of the species used in fourteenth century gut-skin garments from the archaeological site of Nuulliit, Greenland
Source: Sci Rep. 2024 Jun 11;14:13431. doi: 10.1038/s41598-024-63243-0 (PMC11166976; doi:10.1038/s41598-024-63243-0)
Supplement: Supplementary file 1 — Supplementary Information. [file 41598_2024_63243_MOESM1_ESM.docx]

**Supporting Information for**

Paleoproteomic identification of the species used in 14th-century gut-skin garments from the archaeological site of Nuulliit, Greenland

Annamaria Cucina, Anne Lisbeth Schmidt, Fabiana Di Gianvincenzo, Meaghan Mackie, Carla Dove, Aviâja Rosing Jakobsen, Bjarne Grønnow, Martin Appelt, Enrico Cappellini

corresponding authors: Annamaria Cucina (annamariacucina1@gmail.com), Anne Lisbeth Schmidt (anne.lisbeth.schmidt@natmus.dk) and Fabiana Di Gianvincenzo (fdigianvincenzo@palaeome.org)

**This file includes:**

Supporting text

Fig.S1 to S20

**Other SI include:**

Supplementary Table S1 and S2 in Excel format

**Supporting Information Text**

Hereby are reported in more detail the methods used.

**Methods**

*Feather and hair microscopy*

Cross-sections of hair were performed on hair strands embedded as straight and stretched as possible in EPON epoxy resin. Using a microtome, 1 μm thick slices were cut perpendicular to the hair direction. The sections were stained with 1% toluidine blue and mounted on slides in a drop of Petrex with a coverslip over. Hair was washed with acetone for longitudinal mounting, dried, and mounted in Pertex as described above. A Leica DM4 M with transmitted light (TL) (100x, 200x, 400x, and 500x magnification) was used for microscopy. 30-80 hairs from each sample were photographed and evaluated. As references, hairs from species-identified fur skins belonging to the Natural History Museum of Denmark were used. In addition, several DNA identified specimens from the National Museum of Denmark were also used as referential material (34).

Due to the fragile nature of the Nuullit artifacts, small subsamples of feather material were removed from items at the National Museum of Denmark and carefully placed in ziplock bags labeled with the item number.

Minute feather samples were removed from the subsamples and mounted on glass microslides prepared following methods described in Dove and Peurach (2002) (45) at the Smithsonian Institution, Washington, DC USA. The microslides were examined for plumulaceous (downy) barbule characteristics and compared to a large reference collection of known microslides. Feather material was examined microscopically using a Leica© DM750 (Leica Microsystems, Wetzlar, Germany) comparison light microscope at 50x, 100x, 200x, and 400x (power). Photomicrographs were taken with a Leica© DFC290 HD camera (Leica Microsystems, Wetzlar, Germany).

*Proteomic analysis*

Two micro-samples of a historical Greenlandic piece of garment (specimen R), dating back to the 1900’s were removed from the gut skin and the sinew thread, respectively. The samples were analyzed prior to the archeological samples to assess the proteomic approach and compare the ageing process. Furthermore, a laboratory blank was processed in parallel with the sample and analyzed in the same way. Information about the storage of the historical reference is unknown. Therefore, it cannot be ruled out that the garment was treated with biocides. It is however known that the archeological samples were treated with biocides and lipids such as neatsfoot oil and castor oil from conservation treatments (26, 48), and other naturally occurring lipids could affect the analysis. Thus, a protocol for the elimination of compounds interfering with protein extraction was tested on the historical parka, and then applied to the archeological samples. A mixture of hexane/acetone (1:1) was added to each sample, in the volume necessary to submerge the sample completely (<500 uL), heated at 50°C and agitated at 700 rpm for 5 minutes. The samples were then cooled down and centrifuged. The supernatant was removed, and the steps were performed at least three times. This wash procedure was extended for the samples in which the supernatant was still very colored after the third wash. The samples were then dried, and protein residues were extracted from the samples using a lysis buffer as in Mackie et al. (25). In detail, samples were incubated at 80ºC for 2 hours in 100 μL 2M guanidine hydrochloride solution (GndHCl), pH=8.0. The solution was composed of guanidine hydrochloride 2M, TCEP 10mM, CAA 20mM, TRIS 100mM. Proteins were quantified with NanoDrop™ 2000/2000c Spectrophotometers (Thermo Fisher Scientific). A Bradford assay was used as an alternative method (Genesys 10S Vis, Thermo Scientific). Samples were aliquoted to take around 3 µg of proteins, which were digested under agitation at 37ºC in-solution: first with 0.2 μg rLysC (Promega, Sweden) for 2 h; then, after dilution of the GndHCl to a final concentration of 0.6 M using 25 mM Tris in 10% acetonitrile (ACN) in water, with 0.8 μg Trypsin (Promega, Sweden) overnight. Samples were then acidified to pH 2 using 10% TFA. The resulting peptides were immobilized on in-house-made C18 Stage-Tips (49) and desalted using ACN/water (80:20) with TFA 0.1%.

*Mass spectrometry analysis*

Samples were eluted from the stage tips using 30 μL 40% ACN in water into a 96 well MS plate. Using a vacuum centrifuge, the solutions were placed at 40ºC until approximately 3 μL of solution was left. The precipitate was then rehydrated with 5 μL of 0.1% TFA, 5% ACN. Then, samples were separated on a 15 cm column (75 μm inner diameter) in-house laser pulled and packed with 1.9 μm C18 beads (Dr. Maisch, Germany) on an EASY-nLC 1200 (Proxeon, Odense, Denmark) connected to a Q-Exactive HF-X (specimen R) or Exploris 480 (archeological samples) (both instruments Thermo Scientific, Bremen, Germany) on a 77 min gradient. Buffer A was milliQ water. The gradient of eluents used increasing buffer B (80% ACN and 0.1% formic acid), going from 5% to 30% in 50 min, 30% to 45% in 10 min, 45% to 80% in 2 min, held at 80% for 5 min before dropping back down to 5% in 5 min and held for 5 min. Flow rate was 250 nL/min. The column temperature was kept at 40°C (integrated column oven). A wash-blank method using 0.1% TFA, 5% ACN was run in between each sample. The Q-Exactive HF-X was operated in data dependent top 10 mode. Spray voltage was 2 kV, S-lens RF level was set at 50, and heated capillary was kept at 275°C. Over the m/z range 350–1400, full scan mass spectra were recorded at a resolution of 120,000 at m/z 200 with a target value of 3e6 and a maximum injection time of 25 ms. HCD-generated product ions were recorded at a resolution of 60,000 with a maximum ion injection time set to 118 ms and a target value set to 2e5. Normalized collision energy (NCE) was set at 28% and the isolation window was 1.2 m/z with the dynamic exclusion set to 20 s. The Exploris 480 was operated with the same parameters except for the NCE which was done at 30% to account for changes in this parameter measurement made by the manufacturer.


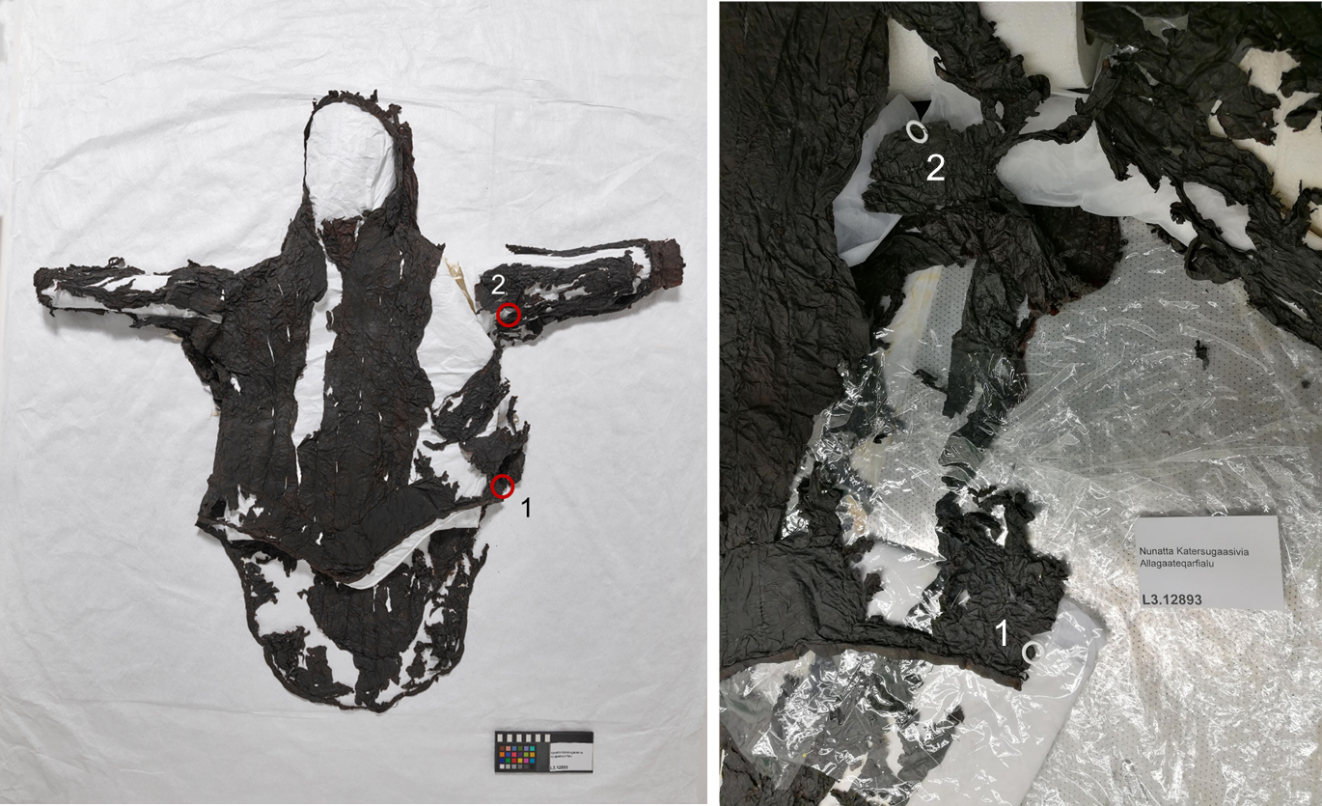


**Fig. S1**. Sampling spots of specimen A: 1) gut skin sample; 2) sinew thread sample. Left: sampling spots on the object. Right: detail.

**
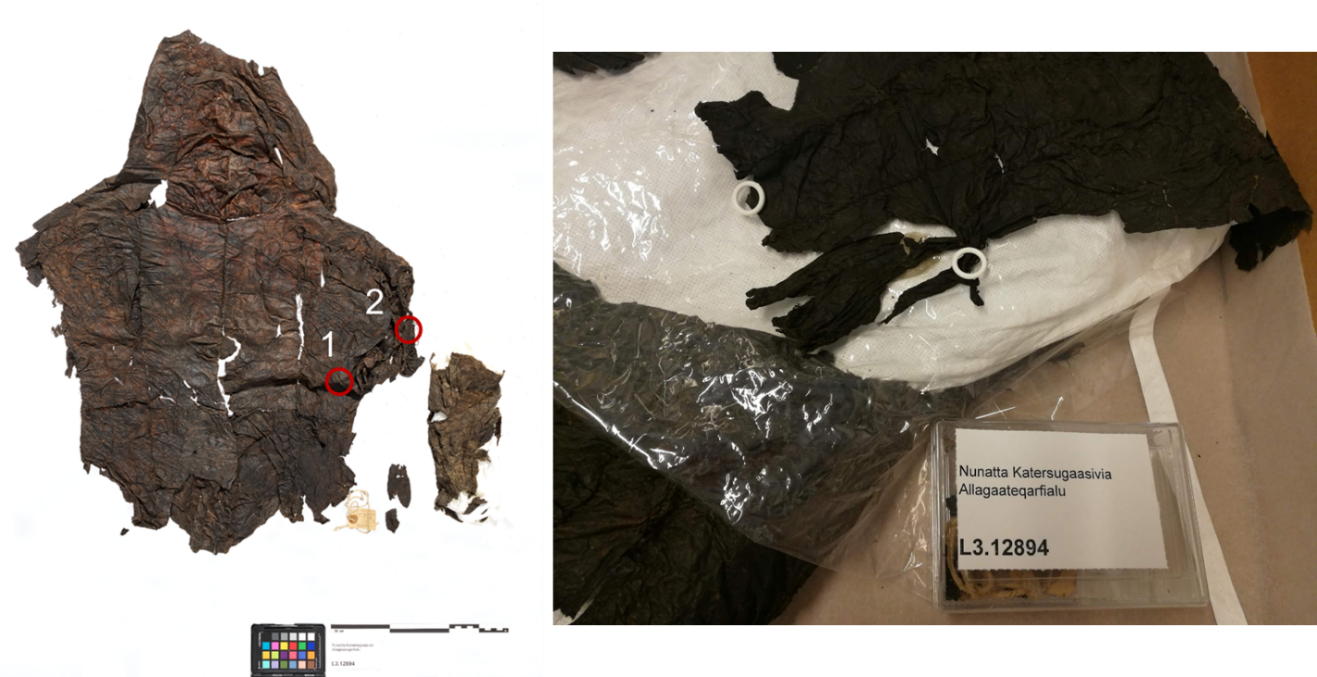
**

**Fig. S2.** Sampling spots of specimen B: 1) gut skin sample; 2) sinew thread sample. Left: sampling spots on the object. Right: detail.


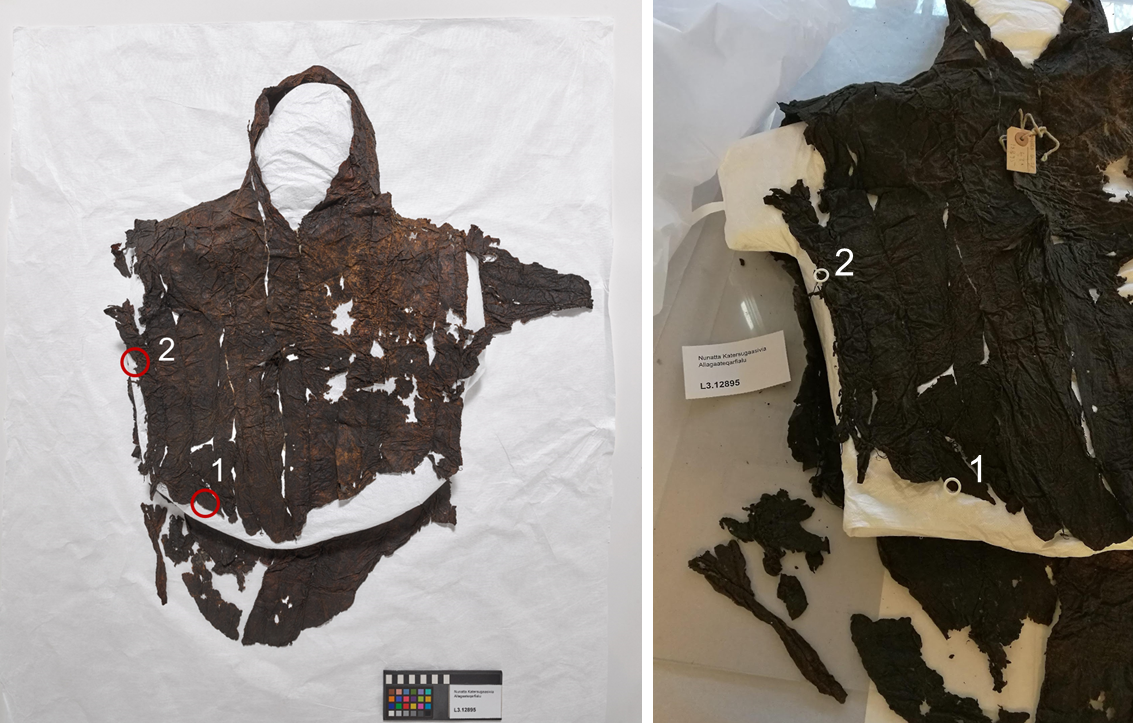


**Fig. S3.** Sampling spots of specimen C: 1) gut skin sample; 2) sinew thread sample. Left: sampling spots on the object. Right: detail.


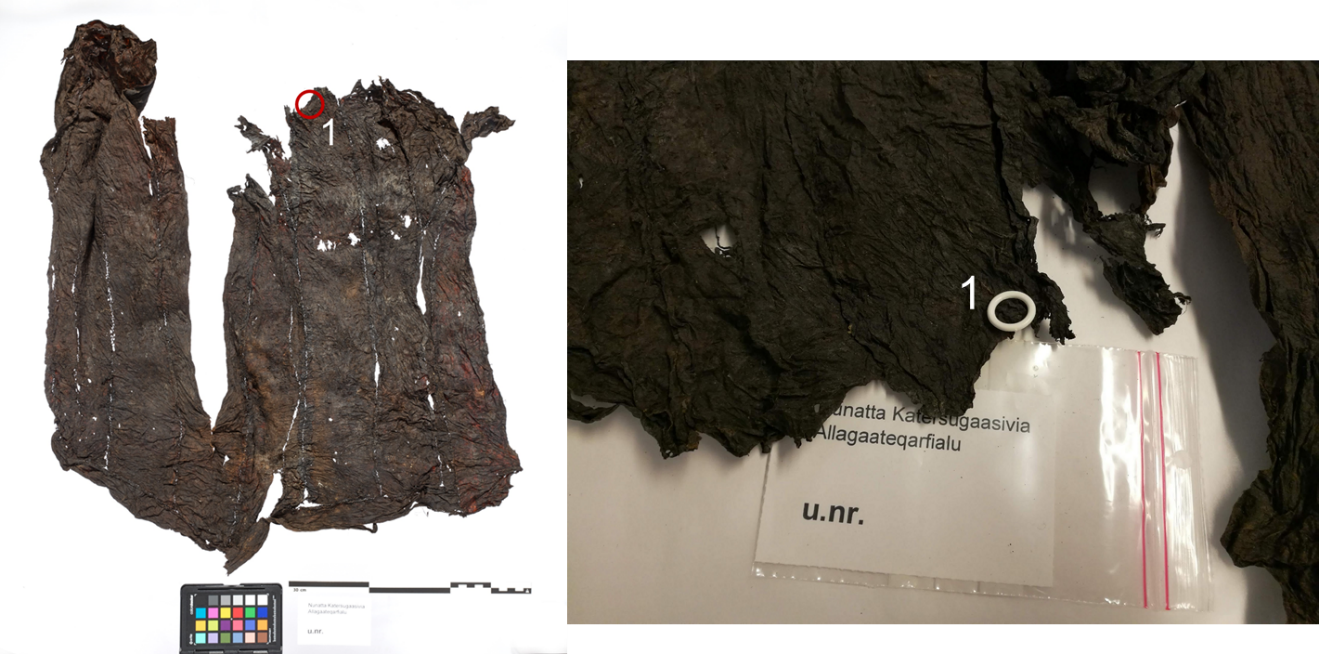


**Fig. S4.** Sampling spot of specimen D: 1) gut skin sample. Left: sampling spot on the object. Right: detail.


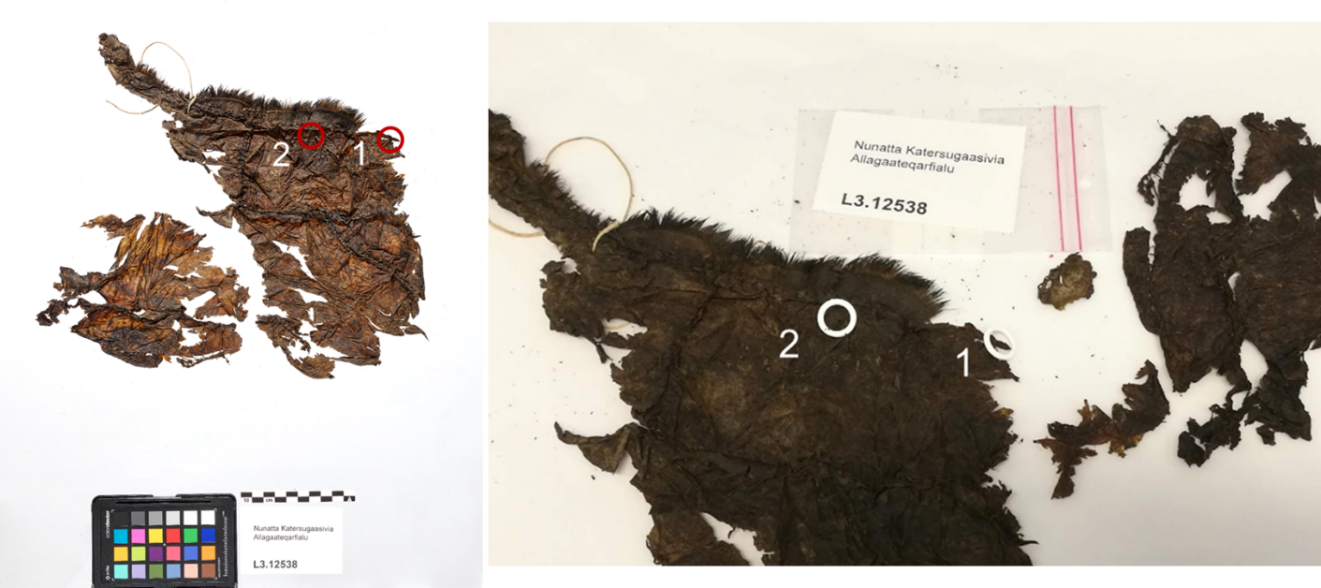


**Fig. S5**. Sampling spots of specimen E: 1) gut skin sample; 2) sinew thread sample.


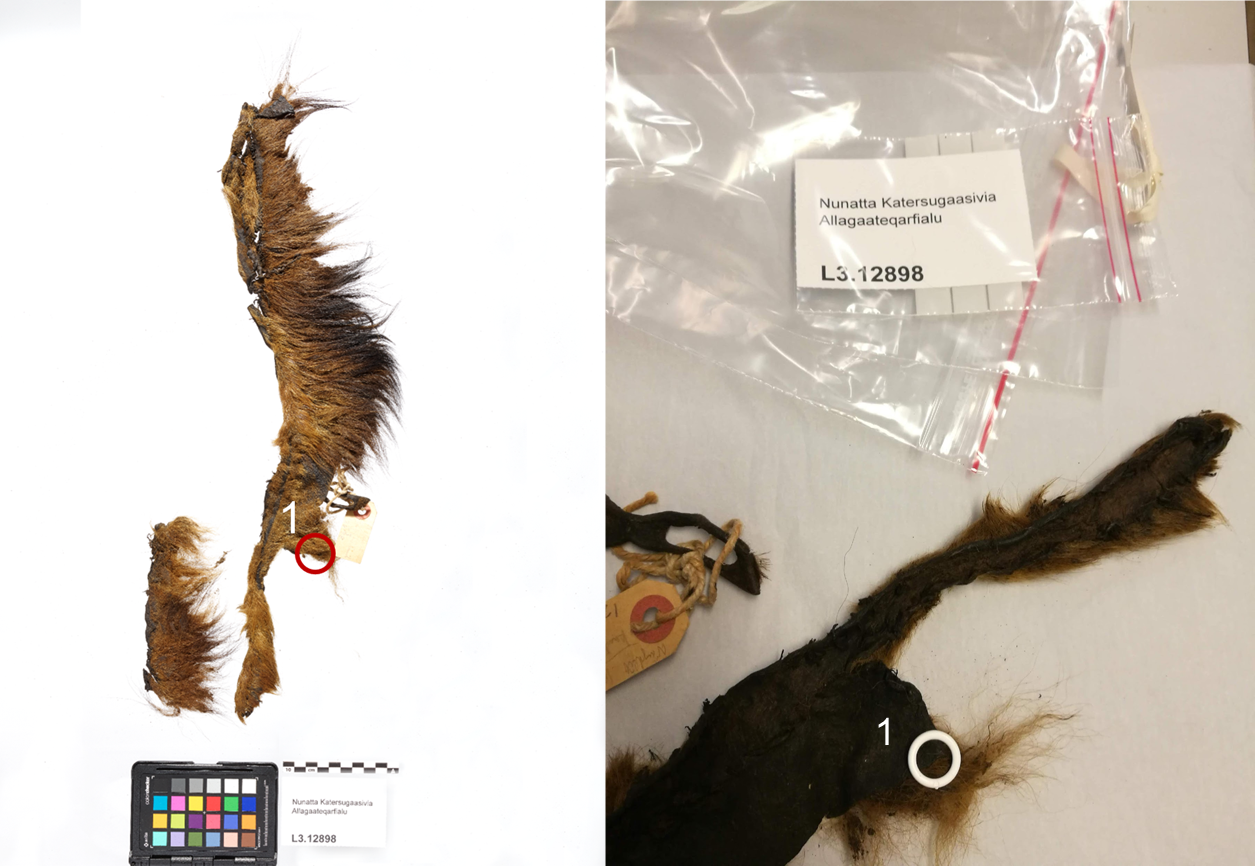


**Fig. S6.** Sampling spot of specimen F: 1) skin and fur sample. Left: sampling spot on the object. Right: detail.


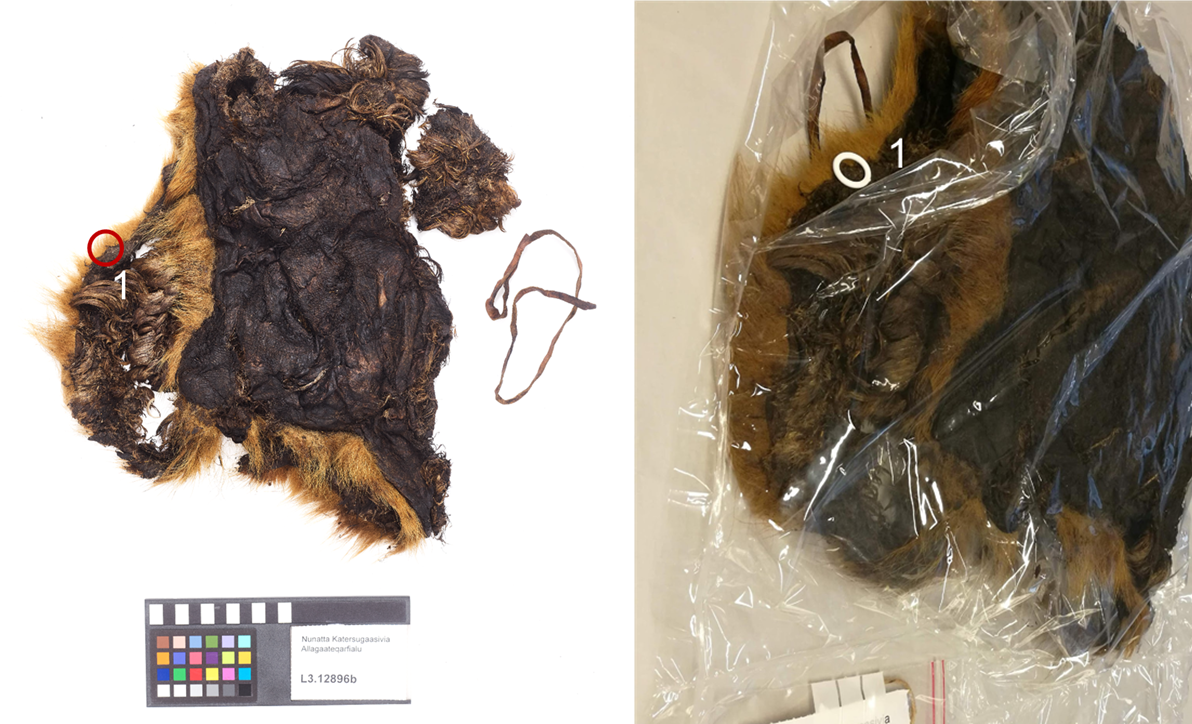


**Fig. S7.** Sampling spot of specimen G: 1) skin and fur sample. Left: sampling spot on the object. Right: detail.


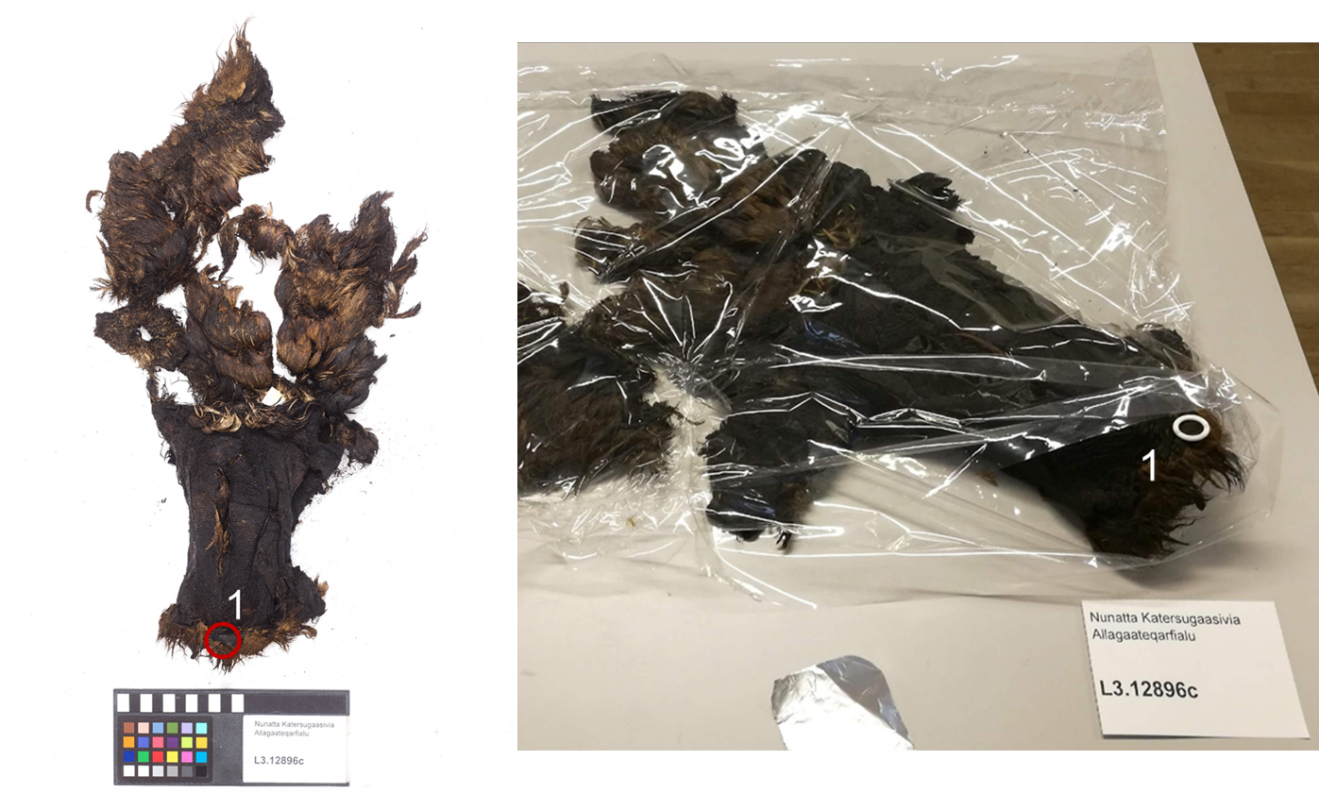


**Fig. S8.** Sampling spot of specimen H: 1) skin and fur sample. Left: sampling spot on the object. Right: detail.


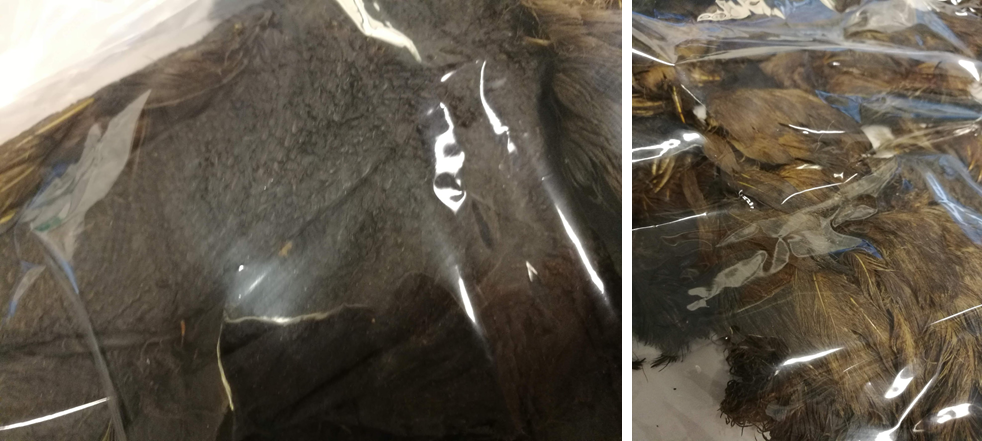
**Fig. S9.** Details of specimen H, showing the visible bird features in the skin and feathers.


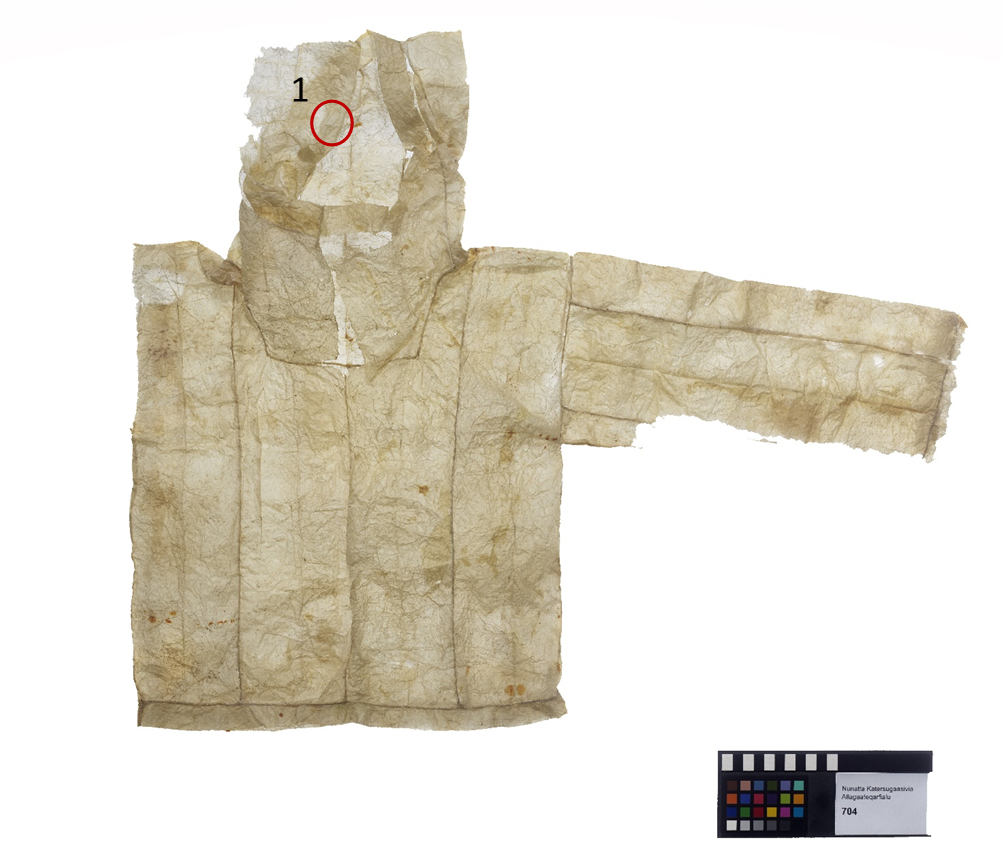


**Fig. S10.** Sampling spot of specimen R: 1) gut skin and sinew thread samples.


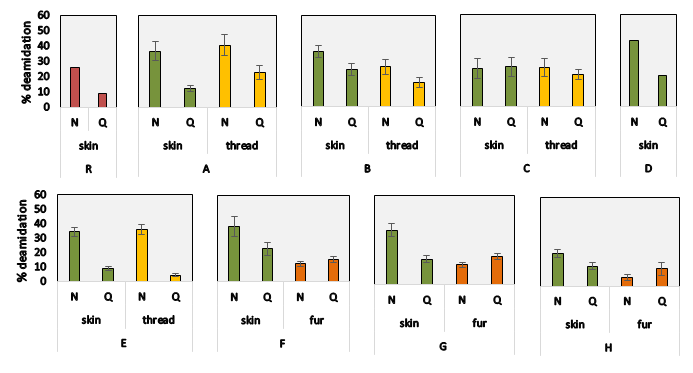


**Fig. S11**. Deamidation rate in skin, thread, and fur samples. The deamidation rate of the historical parka thread was not calculated because of the limited number of peptides (<20 at both residues).


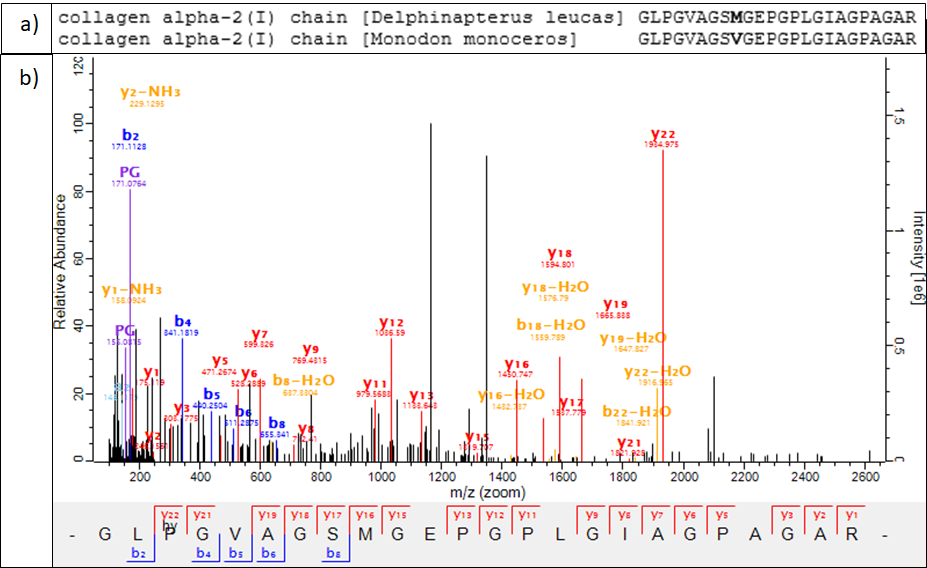


**Fig. S12.** Diagnostic peptide in sample A: a) Alignment of the identified diagnostic peptide of beluga with the sequence of narwhal; b) spectrum of the identified diagnostic peptide of beluga.


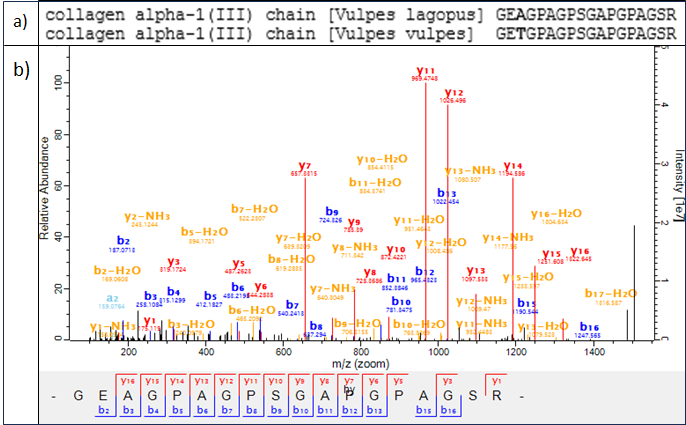


**Fig. S13**. Diagnostic peptide in sample H: a) Alignment of the identified diagnostic peptide of Arctic fox with the sequence of red fox; b). Spectrum of the identified diagnostic peptide of Arctic fox.

| 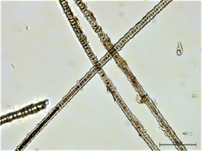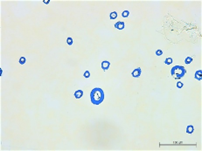 | 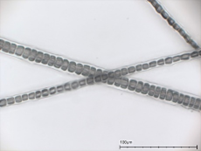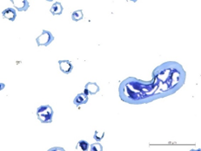 | 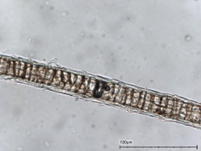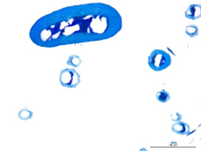 |
| --- | --- | --- |
| Specimen H, trimming on fragmented bird skin garment.  Longitudinal mount: Intermediate hairs with uniserial ladder medulla, medium width. Cross-section: Round to oval primary hairs. Nut shaped intermediate hairs. Medium width medulla with and without remnants. Identified as *V. lagopus.* | Reference: *V. lagopus* CN 2036, adult. Parry? Bay, 1 March 1923 or 28. Whole skin, dorsal sample, The Natural History Museum of Denmark.   Longitudinal mount: Intermediate hair with medium width uniserial ladder medulla. Cross-section: Kidney shaped primary hair, nut shaped intermediate hair. Medium width medullae with remnants. | Reference: *V. lagopus* CN 2357, adult. Naujaat (Repulse Bay), 14 June 1922. Whole skin, dorsal sample. The Natural History Museum of Denmark.   Longitudinal mount: Intermediate hair with medium width uniserial ladder medulla. Cross-section: Oblong primary hair with pigmentation, nut shaped intermediate hair. Medium width medullae with remnants. |

**Fig. S14.** Longitudinal mounted and cross-sectioned *V. lagopus* hairs from specimen H (L3.12896c, fragmented bird skin parka’s trimming) and reference fur skins from The Natural History Museum of Denmark.

####
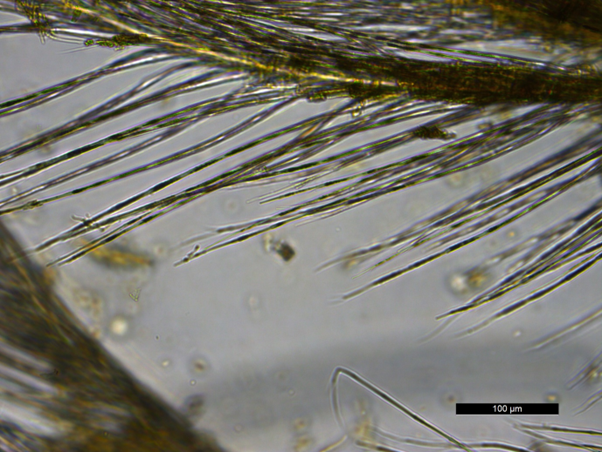


**Fig. S15.** Photomicrograph of specimen G showing short barbules, pigmented nodes and pronged tips typical of Alcidae.

| 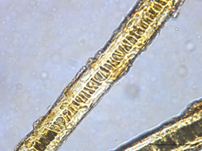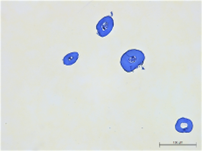 | 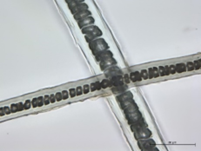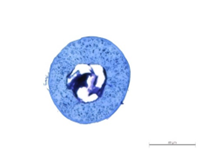 | 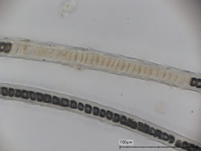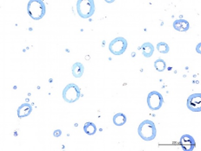 |
| --- | --- | --- |
| Specimen F, top part of woman’s boot. Trimming.  Longitudinal mount: Primary hair with unbroken medulla. Intermediate hair with uniserial ladder medulla, medium width. With and without mounting media.Cross-section: Round to oval primary hairs. Medium width medulla with and without remnants. Intermediate hair without medulla remnants. Probably *C. lupus familiaris.* | Reference: *C. lupus* *familiaris* CN 3294. Adult female, Ittoqqortoormiit (Scoresbysund), 10 January 1928. Whole skin, dorsal sample. Natural History Museum of Denmark.   Longitudinal mount: Intermediate hairs with uniserial ladder medulla, medium width. Cross-section: Round primary hair with cortex pigmentation. Medium width medulla with remnants. | Reference: *C. lupus* CN 4094. Adult male, Umingmak Nuna (Ellesmere Island) 10 November 1953. Whole skin, dorsal sample. Natural History Museum of Denmark.   Longitudinal mount: Intermediate hair with uniserial ladder medulla, with and without mounting media. Medium width medulla. Cross-section: Round to oval primary hairs, many nut shaped intermediate hairs. Medium width medullae with remnants. Few round secondary hairs. |

**Fig. S16.** Longitudinal mounted and cross-sectioned hairs from specimen F (boot shaft trimming) and reference fur skins from The Natural History Museum of Denmark.

| 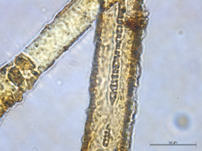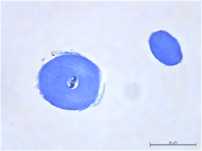 | 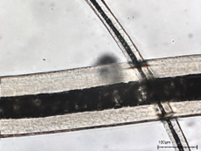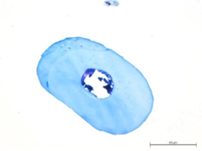 | 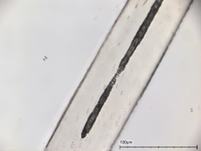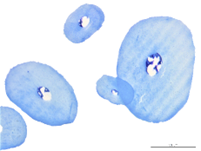 |
| --- | --- | --- |
| Specimen G, trimming on bird skin hood.  Longitudinal mount: Intermediate hair with fragmented uniserial ladder medulla, narrow width. Cross-section: Round primary hair with narrow medulla with remnants. Oval intermediate hair without medulla. Identified as *Ursus maritimus.* | Reference: *Ursus maritimus* M 8009, adult female ZOO Copenhagen 28 November 1970. Whole skin, dorsal sample, The Natural History Museum of Denmark.  Longitudinal mount. Primary hair with unbroken, medium width medulla. Intermediate hair with uniserial ladder medulla. Cross-section. Oval primary hair with sparse cortex pigmentation. Medium width medulla with remnants. | Reference: *Ursus maritimus* M 8009, adult female ZOO Copenhagen 28 November 1970. Whole skin, abdominal sample, The Natural History Museum of Denmark.  Longitudinal mount: Intermediate hair with medium width uniserial ladder medulla.  Cross-section: Oblong primary hair with pigmentation, nut shaped intermediate hair. Medium width medullae with remnants. |

**Fig. S17.** Longitudinal mounted and cross-sectioned *U.maritimus*  hairs from specimen G (trimming on bird skin hood) and reference fur skins from The Natural History Museum of Denmark.


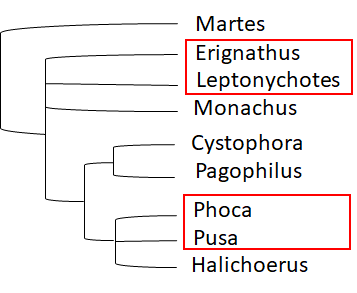

**Fig. S18**. Bootstrap analysis of phylogenetic relationships among seals [(51)](https://paperpile.com/c/7gQlao/ohCWU)**.**


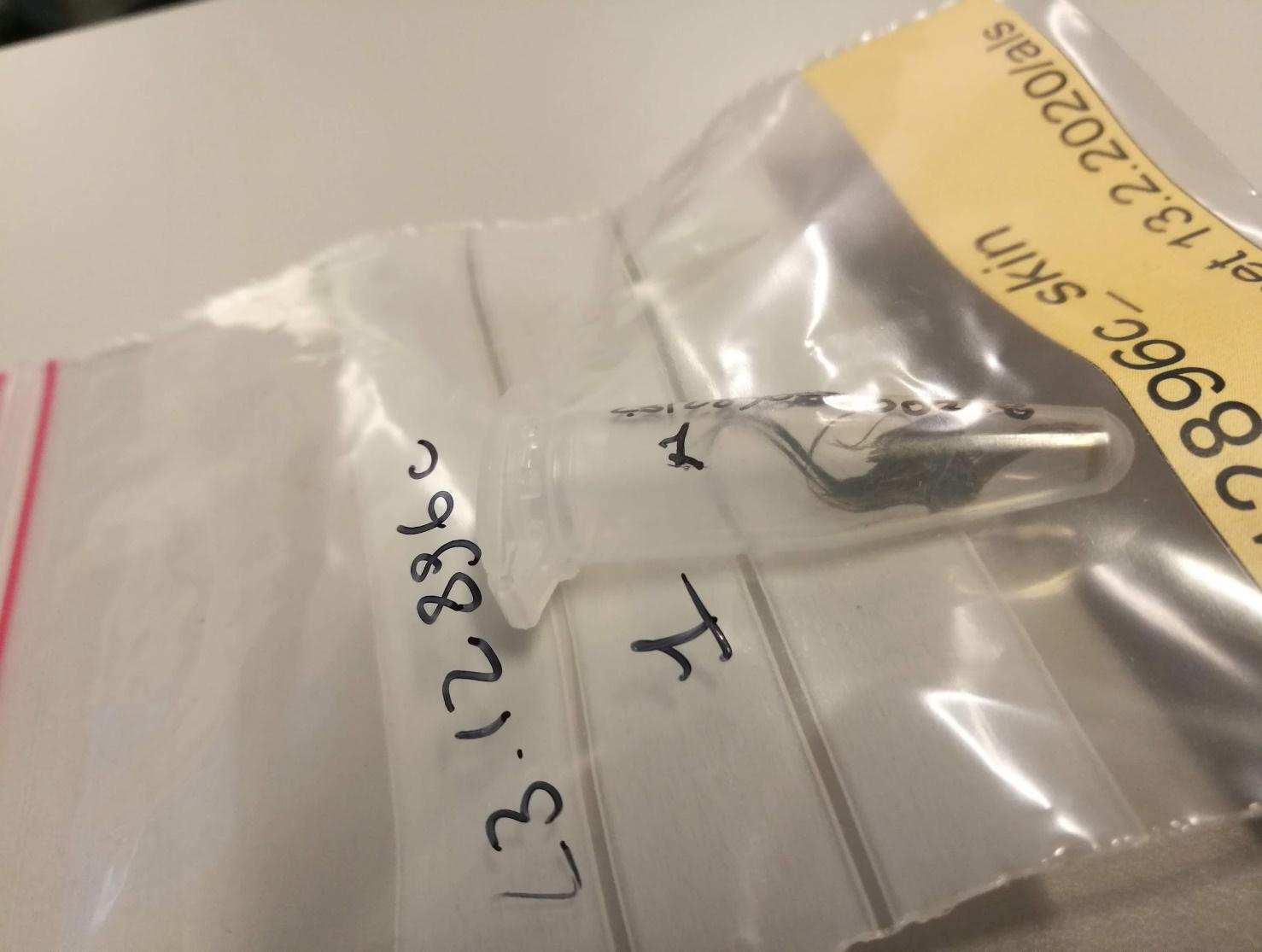


**Fig. S19.** Sample removed from specimen H, before separation into skin and fur.


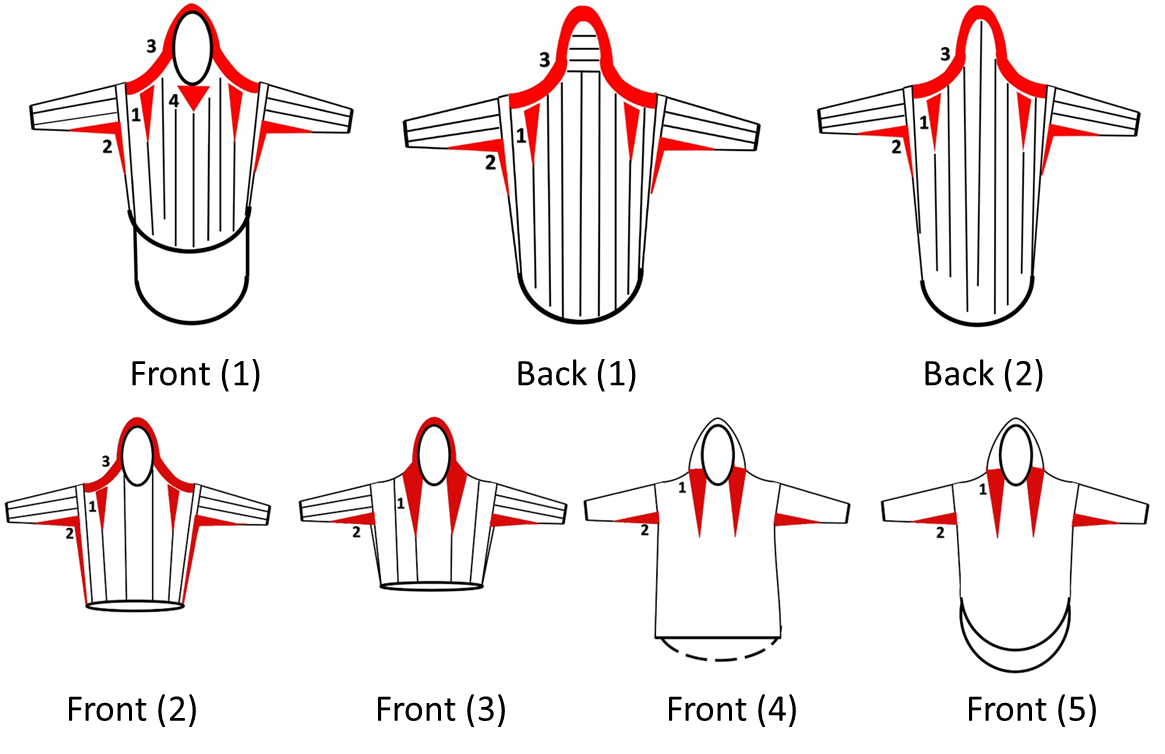


**Fig. S20.** Schemes of sewing patterns in the parkas found in the Nuulliit site. The three Nuulliit gut skin parkas’ 600 year-old-design (*Front (1), Back (1), Back (2)*) include 1) double pointed hood roots inserted in the mid shoulders, 2) gussets under the sleeves and side seams, 3) hood-and-shoulder yoke, and 4) a triangular gusset under the chin. A rounded flap is seen at the lower bottom at the back and front . *Front (1)* was drawn from the front, *Back (1)* and *(2)* from the back. The four examples of Inuit parkas from 1846-1945 (*Front (2), Front (3), Front (4), Front (5)*), drawn from the front, show designs like the Nuulliit parkas with pointed hood roots and sleeve gussets: *Front (2)*: Iñupiat (Alaska) male gut skin parka; *Front (3)*: Iivit (eastern Greenland) male gut skin parka; *Front (4)*: Yupiget (Bering Strait region) and Inuvialuit (Canadian Northwest Territories) male fur skin parkas with straight or slightly curved lower bottoms; *Front (5)*: Inupiat (Alaska) female fur skin parka with curved lower edging at the front and back (flaps). Adapted from [(36)](https://paperpile.com/c/7gQlao/2uRe).
